# Topology-aware hybrid graph-transformer network for Alzheimer's disease diagnosis from structural magnetic resonance imaging

**DOI:** 10.3389/fncom.2026.1834764

**Published:** 2026-07-06

**Authors:** Nadhmi A. Gazem, Shahid Latif, Wad Ghaban, Sultan Noman Qasem

**Affiliations:** 1 Department of Information Systems, College of Business Administration-Yanbu, Taibah University, Medina, Saudi Arabia; 2 School of Computing and Creative Technologies, University of the West of England, Bristol, United Kingdom; 3 Applied College, University of Tabuk, Tabuk, Saudi Arabia; 4 Department of Computer Science, College of Computer and Information Sciences, Imam Mohammad Ibn Saud Islamic University (IMSIU), Riyadh, Saudi Arabia

**Keywords:** Alzheimer's disease diagnosis, deep learning, graph attention networks, MRI, vision transformers

## Abstract

**Introduction:**

Alzheimer's disease (AD) is a progressive neurodegenerative disorder characterized by localized cortical atrophy and large-scale disruption of brain connectivity. Although deep learning (DL) methods have shown promise for neuroimaging-based diagnosis, many approaches fail to jointly capture localized structural changes and global network-level degeneration.

**Methods:**

We propose a topology-aware hybrid DL framework for AD classification from structural MRI. The model integrates (1) a 3D convolutional neural network (CNN) to extract volumetric morphometric features, (2) a dynamic graph attention network (GAT) to infer patient-specific structural connectivity without predefined atlases, and (3) a topology-biased Vision Transformer (Topo-ViT) that incorporates this connectivity into global attention. The framework is trained under strict subject-level data segregation and optimized using focal loss with an AUC-driven strategy.

**Results:**

Evaluated on a structural MRI dataset derived from the OASIS cohort, the proposed model achieved a test ROC-AUC of 0.857, with an overall accuracy of 85% and high sensitivity in detecting demented cases. Ablation studies show that topology-guided attention improves performance over CNN and hybrid baselines. Additional analyses reveal stable connectivity patterns and well-separated latent representations

**Discussion:**

The results demonstrate that integrating topology-aware mechanisms enables more coherent modeling of AD as a network-level disorder. The proposed framework captures both local and global structural patterns, offering improved diagnostic reliability. Further validation on larger datasets is required for clinical deployment.

## 1 Introduction

Alzheimer's disease is a progressive neurodegenerative disorder and the leading cause of dementia worldwide, characterized by gradual cognitive decline, particularly affecting episodic memory in its early stages (Scheltens et al., 2021; Zhao et al., 2023). Alzheimer's disease is increasingly viewed as both a localized neurodegenerative disorder and a large-scale brain network disorder. It is often described as a “disconnection syndrome” (Liu et al., 2023). Pathologically, AD involves region-specific cortical atrophy, particularly in the hippocampus and medial temporal lobe. It also causes widespread disruption of structural and functional brain networks (Ribarič, 2023). Consequently, accurate diagnosis and longitudinal monitoring of structural magnetic resonance imaging (sMRI) data require computational models that capture both localized morphometric alterations and network-level topological changes (NallappaBhavithran and Selvakumar, 2025; Sharma et al., 2022).

Despite the rapid adoption of deep learning (DL) in medical image analysis, several state-of-the-art models still struggle to simultaneously capture multiscale pathological processes (Mohsen, 2025; Slimi et al., 2024; Dhivya et al., 2024). Conventional CNNs are effective at extracting localized, grid-structured features, such as regional cortical thinning or focal atrophy (Zhao et al., 2025). However, their inherently localized receptive fields constrain their ability to model long-range dependencies and large-scale network deterioration characteristic of AD (Yang et al., 2025). Vision Transformers (ViTs) have been introduced to address this limitation by modeling global context through self-attention mechanisms Khatri and Kwon (2023). Nevertheless, standard ViTs lack strong spatial inductive biases and treat images as sequences of independent patches, potentially overlooking the brain's structured anatomical organization and non-Euclidean connectivity patterns (Cong et al., 2024).

Graph Neural Networks (GNNs) offer a promising framework for modeling brain connectivity in non-Euclidean domains (Mienye and Viriri, 2025). However, many existing neuroimaging-based GNN approaches depend on predefined, population-level brain atlases to create static graphs (Ali et al., 2025). Such static models may not sufficiently capture the individualized and progressive aspects of neurodegeneration, in which network topology declines in a patient-specific manner (Vogel et al., 2023). Existing methods struggle to jointly model localized morphometric changes and patient-specific structural connectivity. To address these limitations, we propose a unified, end-to-end differentiable framework for AD diagnosis. To effectively characterize AD as a disconnection syndrome, a computational framework should automatically infer patient-specific network organization and incorporate this structural information into global representation learning. Furthermore, to ensure true clinical reliability, the model must process physical anatomy without disrupting spatial dimensions and must be rigorously protected against data leakage. Therefore, we propose a topologically guided, 3D volumetric hybrid architecture that combines deep convolutional feature extraction, graph-based connectivity learning, and transformer-based global reasoning.

The proposed architecture operates in four sequential stages. First, to preserve inter-slice spatial dependencies and true brain geometry, a truncated 3D convolutional backbone encodes complete sMRI volumes into 3D regional morphometric embeddings. Second, these volumetric embeddings are processed by a dynamic Graph Attention Network (GAT), which moves beyond population-level atlases to learn a patient-specific structural connectivity matrix. Third, this learned graph structure is directly integrated into the self-attention mechanism of a Vision Transformer (Topo-ViT). Instead of attending uniformly across all image patches, the transformer's attention weights are mathematically biased by the learned patient-specific connectivity, thereby guiding global reasoning by the patient's actual physical network deterioration. Finally, the framework employs an AUC-driven diagnostic objective equipped with Focal Loss to actively penalize model errors on hard-to-diagnose, borderline cases typical in imbalanced medical datasets.

The main contributions of this article are summarized as follows:

- **3D Volumetric hybrid architecture:** a unified framework is developed that integrates localized volumetric feature extraction (via 3D CNNs), non-Euclidean connectivity modeling (via GATs), and long-range dependency modeling (via ViTs), specifically tailored to capture the multiscale 3D pathology of AD.
- **Topologically biased self-attention mechanism:** a biologically inspired self-attention formulation is introduced in which dynamically learned patient-specific adjacency matrices modulate the transformer's attention weights. This explicitly penalizes physically unlikely information flow, enabling topology-aware global reasoning guided by structural connectivity priors.
- **Patient-specific network modeling:** the proposed framework eliminates the reliance on static, population-level brain atlases by dynamically learning instance-specific connectivity representations, allowing the model to capture highly individualized trajectories of structural degradation.
- **Rigorous clinical optimization and leakage prevention:** the framework addresses major pervasive pitfalls in medical AI by enforcing strict subject-level data segregation to guarantee zero data leakage between splits. Furthermore, the integration of Focal Loss and an explicitly AUC-driven dynamic learning rate scheduler ensures robust clinical discriminability across heavily imbalanced disease cohorts.

The rest of this article is organized as follows. Section 2 presents an overview of some state-of-the-art DL-based AD detection methods. Section 3 provides a detailed description of the proposed framework. Section 4 details the experimental setup and discussion on outcomes. Finally, Section 5 summarizes the research and outlines future directions.

## 2 Related work

Recent advances in DL have significantly improved the automated diagnosis of AD. However, accurately capturing the complex, multiscale nature of neurodegeneration, ranging from localized structural alterations to large-scale network disconnections, remains a fundamental challenge.

A substantial portion of the literature relies on CNNs operating on 2D MRI slices. Approaches such as the stacked CNN with channel attention (SCCAN) proposed by Hassan et al. (2025), the multiscale spatial attention framework of Tripathy et al. (2024), and the ResNet-50-based model with Grad-CAM interpretability introduced by Gunawan et al. (2025) have demonstrated strong performance in extracting localized morphological features. While these methods are computationally efficient and effective at identifying region-specific biomarkers, their reliance on isolated 2D slices disrupts the inherent volumetric continuity of brain anatomy. As a result, inter-slice dependencies and large-scale structural relationships are not explicitly modeled, limiting their ability to characterize AD as a network-level disorder.

To overcome the locality limitations of CNNs, transformer-based and hybrid architectures have been increasingly adopted. Kadri et al. (2023) combined Swin Transformers, EfficientNet, and CoAtNet for multimodal MRI and PET analysis, achieving strong performance by capturing both local and global dependencies. Similarly, Otapo et al. (2025) integrated MobileViT with TabNet to jointly process MRI slices and clinical data under strict subject-level splitting, improving diagnostic precision. Further enhancements include the bidirectional spatial attention mechanism introduced by Zhao et al. (2026) and the CBAM-enhanced EfficientNet framework proposed by Yildiz and Subasi (2026), which emphasize both feature refinement and rigorous evaluation protocols. Despite these advances, most transformer-based approaches continue to operate on 2D representations or patch-wise tokenization schemes that overlook the brain's true 3D structure. Consequently, their attention mechanisms are primarily driven by semantic similarity rather than by biologically meaningful anatomical connectivity.

More recent studies have begun to explore 3D volumetric modeling and multimodal fusion to better preserve anatomical integrity. Bansal et al. (2026) proposed ClinPatch-AD, which leverages 3D MRI patch tokens alongside clinical data within a transformer framework, leading to improved clinical alignment and prediction ranking. In parallel, Ibad et al. (2026) introduced a CNN-Capsule Network hybrid (NeuroXAI-Caps) to capture pose-equivariant neuroanatomical features and enhance interpretability. While these approaches represent important steps toward richer representations, they either rely on unconstrained global attention or remain limited to 2D inputs, and therefore do not explicitly model patient-specific structural connectivity or large-scale network disintegration.

Overall, three key limitations persist across the existing literature. First, the widespread reliance on 2D slice-based processing fragments the brain's continuous 3D geometry, resulting in the loss of critical volumetric context. Second, although transformer-based models improve global reasoning, their attention mechanisms are typically governed by feature similarity rather than by underlying anatomical pathways. Third, current approaches lack mechanisms to infer patient-specific structural connectivity, thereby preventing them from capturing individualized patterns of neurodegenerative progression.

To address these limitations, we propose a unified, topology-aware framework that integrates three complementary modeling paradigms. Specifically, our approach employs a natively 3D volumetric backbone to preserve anatomical continuity, a dynamic GAT to infer patient-specific connectivity, and a Topo-ViT that incorporates this connectivity directly into global attention. By constraining transformer reasoning with dynamically learned anatomical structures, the proposed model enables biologically coherent and patient-specific diagnostic inference under strict subject-level data segregation.

## 3 The proposed framework

**Figure 1:**
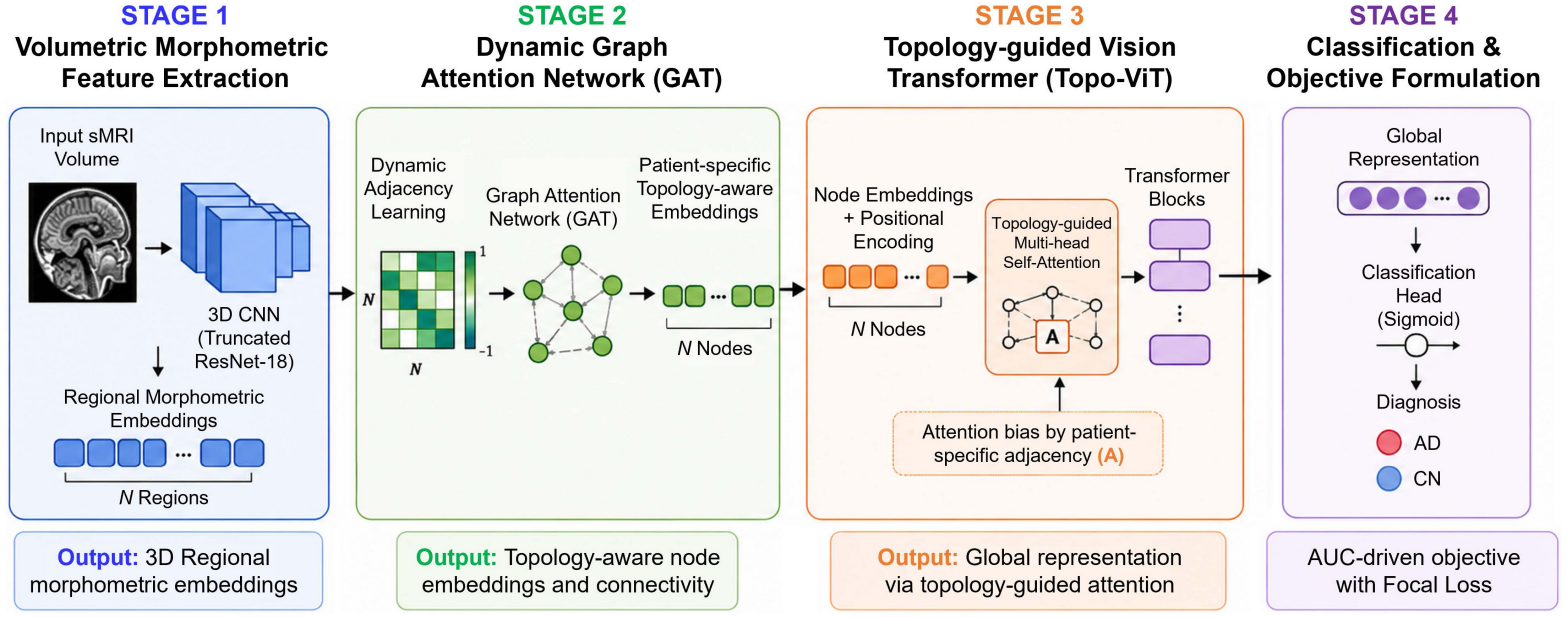
A high-level workflow of the proposed architecture.

A high-level architectural diagram of the proposed design is shown in Figure 1. The proposed framework is designed to explicitly model AD as a structural disconnection syndrome, transitioning from traditional localized analysis to a holistic, network-centric diagnostic approach. The architecture seamlessly integrates localized 3D morphometric feature extraction with global, topology-aware network reasoning. Crucially, to preserve the inherent spatial continuity of the brain and entirely eliminate the risk of data leakage, a pervasive pitfall in medical image analysis, the architecture strictly processes 3D volumetric data under rigorous subject-level splits. The detailed mathematical and procedural workflow of the framework is described in the next sections.

### 3.1 Volumetric morphometric feature extraction

The initial phase of the architecture is dedicated to identifying localized neuroanatomical alterations, such as focal cortical thinning and ventricular enlargement. Unlike conventional slice-based approaches that process individual MRI slices independently, thereby losing the three-dimensional anatomical context, our framework uses a natively 3D convolutional backbone. This ensures that the intricate inter-slice spatial dependencies and the brain's true volumetric geometry are preserved throughout the feature extraction process.

Given a preprocessed input structural MRI volume *X*∈ℝ^*C*×*D*×*H*×*W*^, where *C, D, H*, and *W* denote the channel depth, volumetric depth (number of slices), spatial height, and spatial width dimensions, respectively, we employ a truncated 3D ResNet-18 architecture as the foundational feature extractor. Deep 3D networks are notoriously prone to overfitting on constrained medical datasets due to their massive parameter counts. To mitigate this, the early layers of our network are explicitly frozen during training. These frozen layers act as generalized volumetric edge and texture detectors, while only the deeper layers are fine-tuned to capture AD-specific pathological patterns.

The output of the truncated 3D convolutional module is a high-dimensional spatial feature map. To standardize the variable sequence length for the downstream graph and transformer modules while preserving a coarse structural geometry, an adaptive 3D average pooling operation is applied. This yields a uniform volumetric feature map $M\in {\mathbb{R}}^{d_{cnn}\times D^{\prime }\times H^{\prime }\times W^{\prime }}$, where *d*_*cnn*_ is the channel depth, and the spatial dimensions are compressed to a fixed *D*′ = 4, *H*′ = 4, and *W*′ = 4.

These volumetric spatial dimensions are subsequently flattened to generate a sequence of regional feature nodes $V\in {\mathbb{R}}^{N\times d_{cnn}}$, where *N* = *D*′ × *H*′ × *W*′ = 64 represents the total number of distinct 3D anatomical macro-patches. A linear projection matrix $W_{\text{proj}}\in {\mathbb{R}}^{d_{\text{cnn}}\times d_{\text{model}}}$ compresses these highdimensional features to a standard model embedding dimension, *d*_model_. Finally, to guarantee that the permutation-invariant graph and transformer layers retain an explicit understanding of the original 3D anatomical coordinates of each patch, a learnable positional embedding $P\in {\mathbb{R}}^{N\times d_{\text{model}}}$ is added to the projected nodes. The resulting spatially aware node embeddings, $H^{\left(0\right)}\in {\mathbb{R}}^{N\times d_{\text{model}}}$, serve as a robust foundation for subsequent connectivity analysis. The complete procedural workflow for this volumetric extraction process is formally summarized in Algorithm 1.

Algorithm 1 Volumetric Morphometric Feature Extraction 
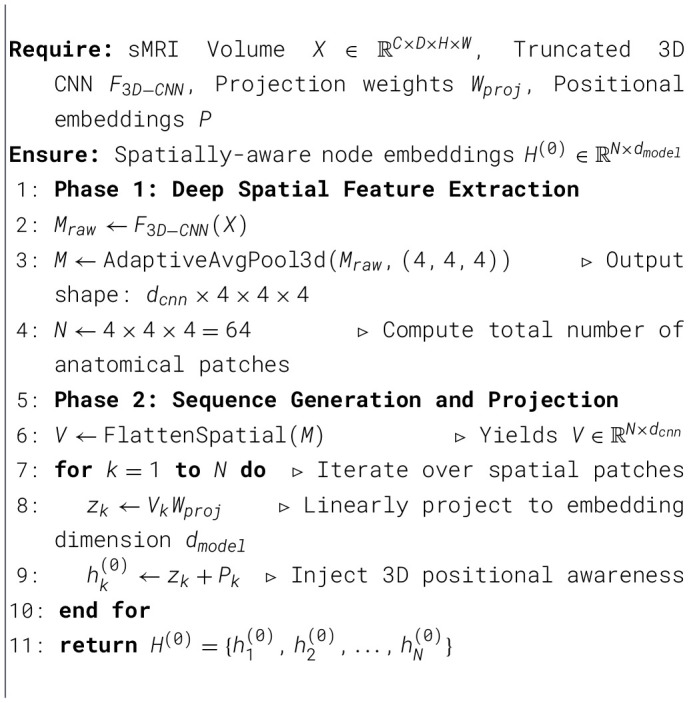

### 3.2 Dynamic graph attention for patient-specific connectivity

A significant limitation of existing graph-based diagnostic models is their reliance on static, population-level brain atlases. Because the trajectory of neurodegeneration and the ensuing structural network deterioration vary significantly across individual patients, generic atlases fail to capture personalized pathological patterns. To overcome this, we implement a GAT that dynamically infers a patient-specific structural connectivity network directly from the extracted latent features.

The GAT module receives the spatially-aware nodes *H*^(0)^ and computes an adaptive adjacency matrix *A*∈ℝ^*N*×*N*^. This matrix represents the individualized strength of structural connectivity between all pairs of the 64 anatomical macro-patches. For any two nodes *h*_*i*_ and *h*_*j*_, a shared linear transformation parameterized by $W_{g}\in {\mathbb{R}}^{d_{\text{model}}\times d_{\text{model}}}$ is applied. A self-attention mechanism, utilizing a trainable weight vector $a\in {\mathbb{R}}^{2d_{\text{model}}}$, calculates the raw, unnormalized connectivity score *e*_*ij*_ as defined in Equation 1:


$$\begin{array}{l} e_{ij}=\mathrm{LeakyReLU}\left(a^{T}\left[W_{g}h_{i}||W_{g}h_{j}\right]\right) \end{array}$$ (1)

where || denotes the concatenation operation. These raw scores indicate the structural relevance of node *j* to node *i*. To ensure numerical stability and to make the coefficients readily comparable across different regional neighborhoods, the scores are normalized using the softmax function. This forms a valid probability distribution over neighboring regions, yielding the final dynamic adjacency matrix as shown in Equation 2:


$$\begin{array}{l} A_{ij}=\frac{\exp\left(e_{ij}\right)}{\overset{N}{\underset{k=1}{\sum }}\exp\left(e_{ik}\right)} \end{array}$$ (2)

Once the patient-specific network topology is established, a graph convolutional message-passing step is executed. The features of each node are updated by aggregating the linearly transformed features of all other nodes, heavily weighted by the learned connectivity strengths. The updated, topologically enriched node embeddings *H*^(1)^ are computed via Equation 3:


$$\begin{array}{l} H^{\left(1\right)}=\mathrm{ReLU}\left(A\left(H^{\left(0\right)}W_{g}\right)\right) \end{array}$$ (3)

This graph-based aggregation effectively links localized atrophy to a global connectivity framework, highlighting both preserved structural pathways and those degraded by AD. The detailed computational steps for this dynamic, patient-specific graph construction are encapsulated in Algorithm 2, which seamlessly processes the 64 volumetric patches defined in the previous phase.

Algorithm 2 Dynamic Patient-Specific Graph Construction 
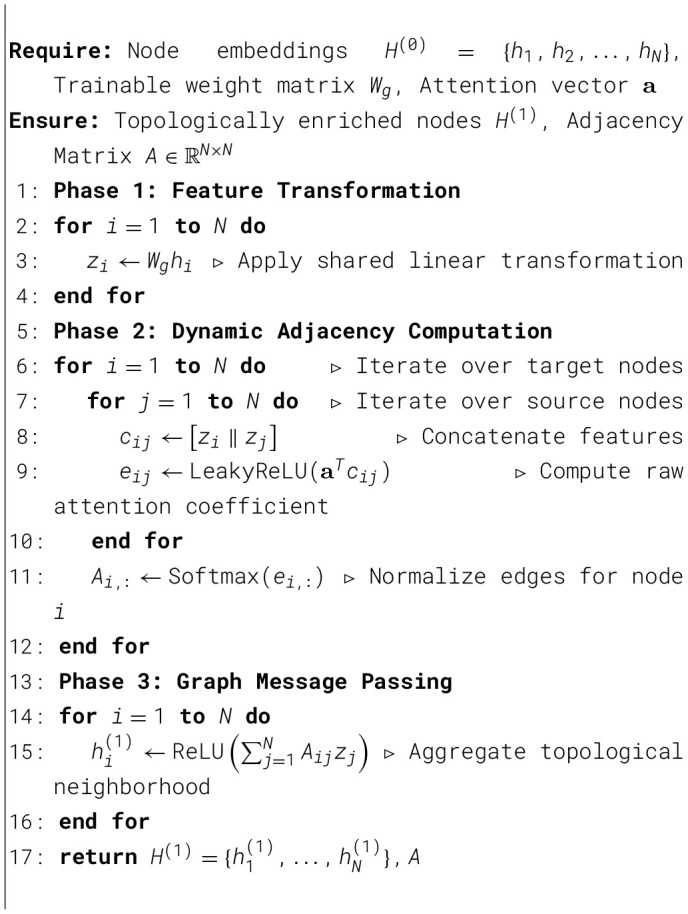

### 3.3 Topologically Biased vision transformer (Topo-ViT)

While the dynamic GAT is highly effective at mapping patient-specific structural pathways, modeling the macro-level cognitive decline associated with AD requires sophisticated reasoning over long-range, global correlations. Standard ViTs natively provide this via dense multi-head self-attention mechanisms. However, standard self-attention operates purely on semantic feature similarity and is completely blind to physical anatomical constraints.

To bridge this gap, we introduce a core algorithmic innovation: we inject the dynamic adjacency matrix *A* directly into the Transformer's attention mechanism, thereby introducing a powerful “topological bias”. This forces the Transformer to evaluate global disease patterns strictly through the lens of the patient's physically degraded structural network.

The topologically enriched nodes *H*^(1)^ are linearly projected into Query (*Q*), Key (*K*), and Value (*V*) matrices. In the proposed Topologically Biased Multi-Head Attention (TBA) mechanism, the patient-specific network *A* is added directly as a spatial bias term to the pre-softmax logits, mathematically formulated in Equation 4:


$$\begin{array}{l} \mathrm{TBA}\left(Q,K,V,A\right)=\mathrm{Softmax}\left(\frac{QK^{T}}{\sqrt{d_{k}}}+\gamma A\right)V \end{array}$$ (4)

where *d*_*k*_ is the scaling factor denoting the dimensionality of the keys, and γ∈ℝ is a learnable scalar weight that dynamically modulates the structural influence on the global reasoning process. Clinically, if the GAT determines that a specific pathway between two brain regions is severely deteriorated due to AD pathology, the corresponding entry in matrix *A* approaches zero. Consequently, the Topo-ViT is mathematically penalized for attending to that “disconnected" pathway, drastically reducing pathological noise and ensuring biologically coherent feature extraction.

Furthermore, transformer architectures can easily overfit on small sample sizes. To prevent vanishing gradients and maintain regularization, the Topo-ViT block incorporates a highly specific sequence of residual connections, Layer Normalization (LN), and a Feed-Forward Network (FFN) equipped with aggressive dropout ( ρ = 0.4 ), expressed in Equations 5, 6:


$$\begin{array}{l} H_{\text{res}}=\text{L}{\text{N}}_{1}\left(H^{\left(1\right)}+\mathrm{Dropout}\left(\mathrm{TBA}\left(H^{\left(1\right)},A\right)\right)\right) \end{array}$$ (5)

$$\begin{array}{l} H^{\left(2\right)}=\text{L}{\text{N}}_{2}\left(H_{\text{res}}+\mathrm{Dropout}\left(\mathrm{FFN}\left(H_{\text{res}}\right)\right)\right) \end{array}$$ (6)

The detailed execution flow of this biologically inspired, topologically biased multi-head attention mechanism is presented in Algorithm 3.

Algorithm 3 Topologically Biased Multi-Head Attention (Topo-ViT) 
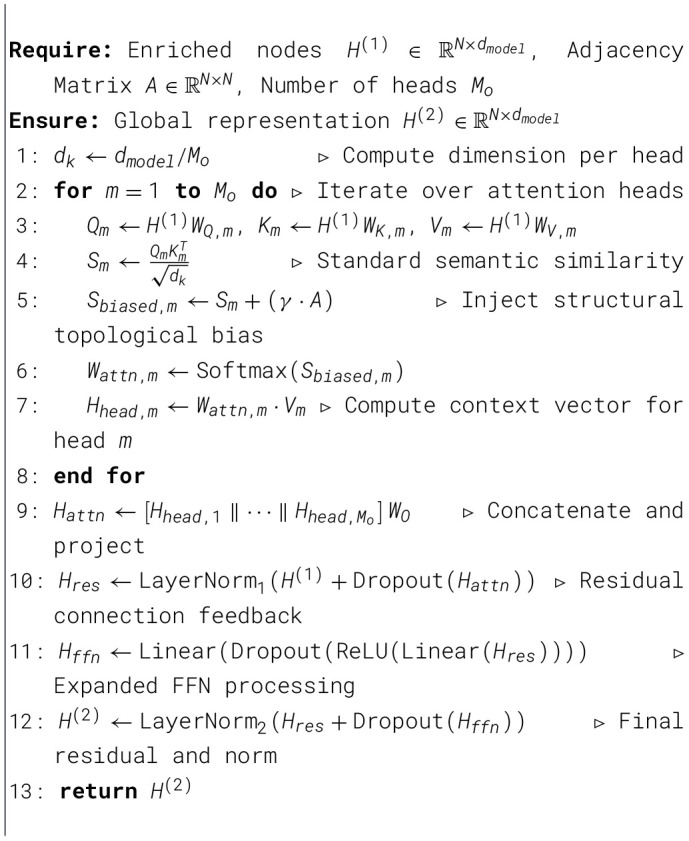

### 3.4 Classification and objective formulation

Following the extraction of the topology-biased global representations, the network aggregates the sequence to yield a final diagnostic prediction. Global Average Pooling (GAP) is applied across the node sequence *H*^(2)^, collapsing the spatial dimensions to result in a singular, unified latent embedding $z_{\text{global}}\in {\mathbb{R}}^{d_{\text{model}}}$ that summarizes the global brain state, as defined in Equation 7:


$$\begin{array}{l} z_{\text{global}}=\frac{1}{N}\overset{N}{\underset{i=1}{\sum }}H_{i}^{\left(2\right)} \end{array}$$ (7)

This feature vector is passed through a multi-layer perceptron dense classifier block formulated as *u* = Dropout(ReLU(*W*_*c*1_*z*_global_+*b*_*c*1_)). Finally, the raw, unnormalized logits *L* = *W*_*c*2_*u*+*b*_*c*2_ are used to determine the diagnostic class. A critical addition to this classifier is the dynamic initialization of the final bias weights *b*_*c*2_. In imbalanced medical datasets, models frequently fall into a local minimum early in training by blindly predicting the majority class (e.g., “Non Demented”). To address this, the initial bias is calculated algebraically based on the class prevalence ratio of the training set ( *b*_initial_ = ln ( pos_ratio/neg_ratio )), ensuring immediate, stable convergence.

**Focal loss optimization:** focal Loss Optimization: Standard categorical cross-entropy fails to effectively penalize errors on minority classes and treats all examples equally, making it highly susceptible to severe class imbalance. Differentiating between healthy aging and early-stage AD is notoriously difficult, yet crucial. We therefore formulate our training objective using Focal Loss (ℒ_*FL*_). Focal loss introduces a modulating factor that dynamically scales the loss based on prediction confidence, heavily penalizing the model for misclassifying difficult, borderline cases, while down-weighting the loss contribution from easily diagnosed examples.

For a target clinical class label *t*, let *p*_*t*_ denote the model's estimated probability for that exact class. The loss is formulated in Equation 8:


$$\begin{array}{l} {ℒ}_{FL}=-\alpha_{t}{\left(1-p_{t}\right)}^{\gamma }\log\left(p_{t}\right) \end{array}$$ (8)

where α_*t*_ is the computed inverse frequency weight for class *t* (handling gross dataset imbalance), and γ = 2.0 is the focusing parameter (handling sample difficulty). The entire framework is trained end-to-end via backpropagation to minimize this focal objective.

Furthermore, to maintain stable parameter updates, gradients are accumulated over multiple steps and clipped to a maximum norm. Crucially, rather than relying on basic loss minimization for model selection, the learning rate scheduling and early stopping mechanisms are strictly driven by the validation set's Receiver Operating Characteristic Area Under the Curve (ROC-AUC) score. This guarantees that the final selected model is optimized strictly for robust clinical discriminability across all diagnostic thresholds. This complete training process is formalized in Algorithm 4.

Algorithm 4 End-to-End Training with AUC-Driven Dynamic Feedback 
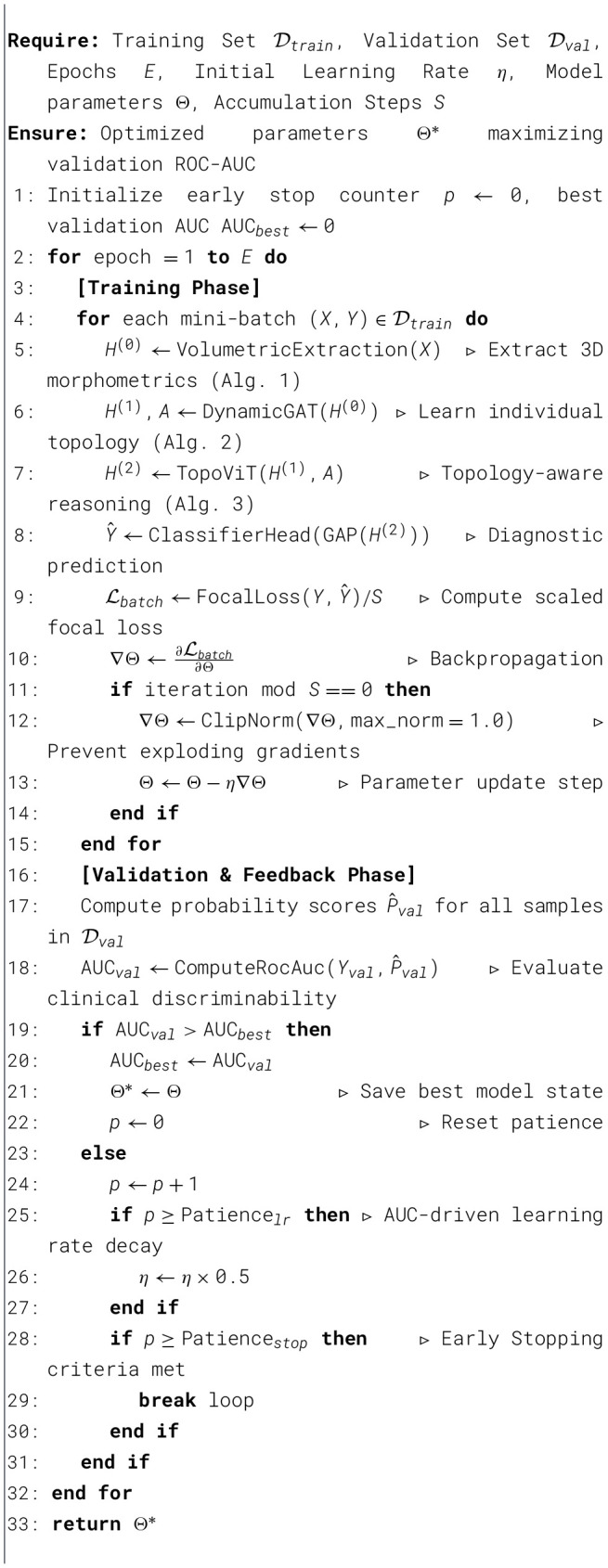

## 4 Experiments and results

This section presents a detailed evaluation of the proposed topology-aware hybrid graph-transformer network. The discussion begins by detailing the experimental setup, hardware specifications, and the characteristics of the utilized structural MRI dataset. Subsequently, standardized data preprocessing and subject-level splitting protocols, designed to strictly prevent data leakage, are outlined. Finally, an analysis of the model's diagnostic performance and computational efficiency is provided, followed by a thorough ablation study to validate the individual contributions of the proposed architectural components.

### 4.1 Experimental setup

The proposed architecture was implemented, trained, and evaluated in the Google Colab Pro environment, accelerated by an NVIDIA T4 Tensor Core GPU, to handle the computational and memory requirements of processing 3D volumetric neuroimaging data. The core DL architecture was developed using the PyTorch framework, leveraging Torchvision to instantiate the pre-trained 3D convolutional backbone and to execute volume-consistent spatial transformations. For rigorous computation of clinical evaluation metrics such as the Receiver Operating Characteristic Area Under the Curve (ROC-AUC) and the Matthews Correlation Coefficient (MCC), Scikit-learn was used exclusively. Additionally, NumPy, Matplotlib, and Seaborn were used for latent-space manipulation and for generating high-fidelity performance visualizations.

To ensure smooth processing and reproducibility across the multi-stage network, the system's hyperparameters were carefully selected. The configuration, encompassing the input data's spatial dimensions, 3D convolutional feature extraction layers, dynamic graph attention mechanisms, vision transformer blocks, and the focal loss-based optimization strategy, is detailed in Table 1.

**Table 1:** The utilized hyperparameters for the proposed architecture.

Component	Hyperparameter	Value
Input data	Spatial resolution	112 × 112
	Volumetric Depth (Slices per patient)	32
3D CNN backbone	Architecture	Truncated 3D ResNet-18
	Frozen Layers	Layers 0 through 2 (Stem, Layer 1, Layer 2)
	Output Pooling Dimensions (*D*′ × *H*′ × *W*′)	4 x 4 x 4
	CNN Feature Dimension (*d*_*cnn*_)	512
	Number of Regional Patches (*N*)	64
GAT & Topo-ViT	Model Embedding Dimension (*d*_*model*_)	256
	Attention Heads	4
	Head Dimension (*d*_*k*_)	64
	Feed-Forward Network (FFN) Dimension	512
	Topo-ViT Dropout (ρ)	0.4
	GAT LeakyReLU Negative Slope	0.2
Classifier head	Dense Layer Dimension	128
	Classifier Dropout	0.5
	Output Classes	2 (Binary Classification)
Optimization & training	Loss Function	Focal Loss
	Focal Loss Focusing Parameter (γ)	2.0
	Optimizer	Adam
	Base Learning Rate (η)	5e-5
	Weight Decay (L2 Regularization)	1e-3
	Batch Size	4
	Gradient Accumulation Steps	4
	Max Gradient Norm	1.0
	Total Training Epochs	50
	Learning Rate Scheduler	ReduceLROnPlateau (Factor: 0.5, Patience: 2)

### 4.2 Dataset description

The primary dataset used to evaluate the proposed framework is derived from the Open Access Series of Imaging Studies (OASIS) MRI dataset (Marcus et al., 2007), a publicly available repository of brain MRI scans. The main objective of this dataset is to facilitate advanced research into the early morphological signs of Alzheimer's disease progression. Ground-truth patient classification was strictly based on the provided clinical metadata and the established Clinical Dementia Rating (CDR) scores. This categorization segments the dataset into distinct stages of disease progression, enabling the nuanced study of morphological changes across varying levels of cognitive decline.

To make the raw imaging data compatible with modern DL pipelines, an data extraction and conversion process was performed. The original medical imaging files were systematically converted into a universally standardized format using the FMRIB Software Library (FSL). Following this, a targeted subset of 461 distinct subjects was extracted and fully processed. To construct the volumetric inputs required for the 3D neural network, each patient's 3D brain scan was sectioned along the z-axis into 256 continuous slices. To optimize computational efficiency while preserving the relevant anatomical structures, an axial window spanning slices 100–160 was isolated for each patient.

**Figure 2:**
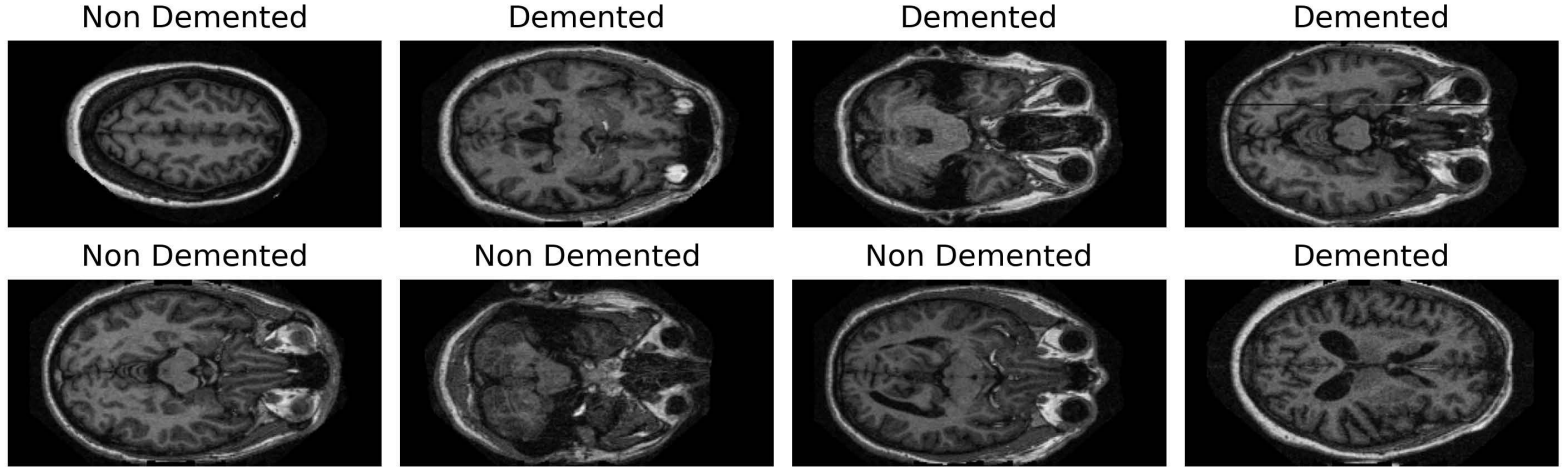
Representative structural MRI slices comparing healthy and Alzheimer's disease cohorts.

The final standardized slices were converted into image files, yielding a curated, easily accessible dataset with an approximate storage footprint of 1.3 GB. Representative axial cross-sections from this processed dataset, highlighting structural variations between diagnostic cohorts, are shown in Figure 2.

### 4.3 Dataset preprocessing and training process

#### 4.3.1 Subject-level data splitting

A known methodological limitation in the existing medical imaging literature is 2D slice-level splitting, in which individual slices from the same patient's brain volume inadvertently appear in both the training and testing datasets. This can compromise model evaluation by inducing data leakage, resulting in artificially inflated performance metrics that do not generalize to clinical settings. To address this issue, a strict subject-level splitting protocol was enforced across the entire dataset. Data were allocated so that all 32 sequential slices for a single patient were assigned exclusively to a single split (training, validation, or testing).

**Figure 3:**
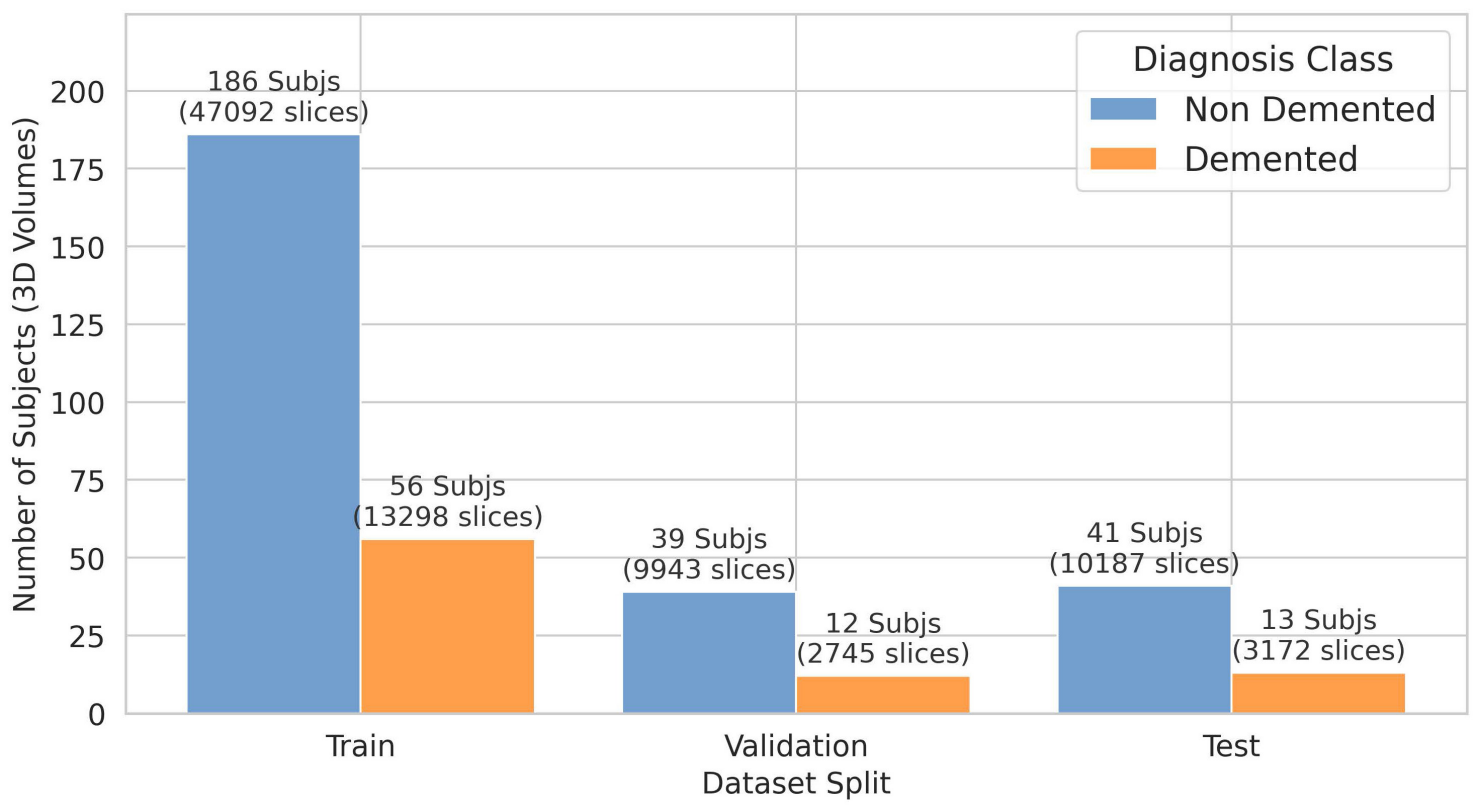
Subject-level dataset distribution across training, validation, and testing splits, ensuring zero data leakage.

While the original dataset cohort comprised 461 subjects, the final dataset comprised 347 subjects. This reduction was a necessary consequence of rigorous quality control; subjects were excluded if they possessed corrupted sequence files, ambiguous clinical metadata, or an insufficient number of continuous axial slices required to construct a full 3D volumetric tensor. The final distribution of these 347 subjects, reflecting the inherent class imbalance of real-world clinical scenarios, is illustrated in Figure 3. This strict segregation guarantees that the testing phase evaluates the architecture's genuine diagnostic capabilities on entirely unseen anatomical geometries.

#### 4.3.2 Volume-consistent 3D preprocessing

Following the subject-level allocation, neuroimaging data underwent a standardized preprocessing pipeline to optimize inputs for the 3D convolutional backbone. Initially, the high-resolution raw images were uniformly resized to 112 × 112 pixels, thereby reducing the computational burden while preserving critical morphological features.

**Figure 4:**
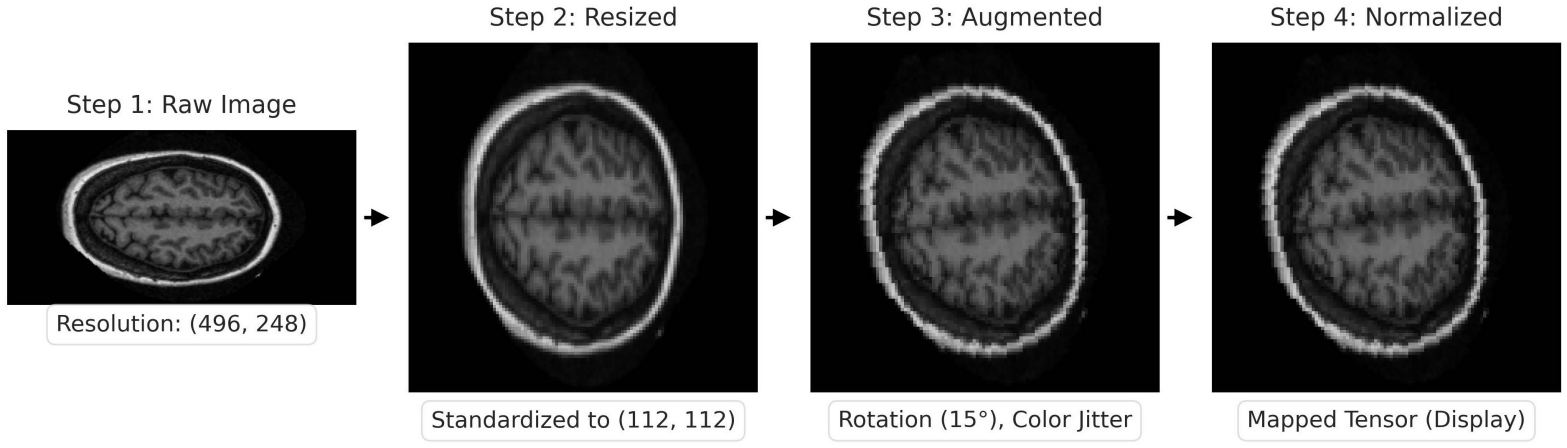
Sequential volumetric preprocessing stages including resizing, consistent 3D augmentation, and tensor normalization.

During the training phase, data augmentation was applied to enhance the model's spatial invariance and robustness against scanning artifacts. Crucially, rather than augmenting each 2D slice independently, which would geometrically distort the brain and destroy the 3D spatial continuity, a volume-consistent transformation strategy was implemented. A single randomized set of augmentation parameters, including rotations up to 15 degrees and color jitter for brightness and contrast, was generated and applied uniformly across all 32 slices of a given patient's volume. Finally, the augmented volumes were converted to PyTorch tensors and normalized using the established mean and standard deviation. This sequential pipeline is visually summarized in Figure 4.

#### 4.3.3 Hierarchical feature extraction and training progression

**Figure 5:**
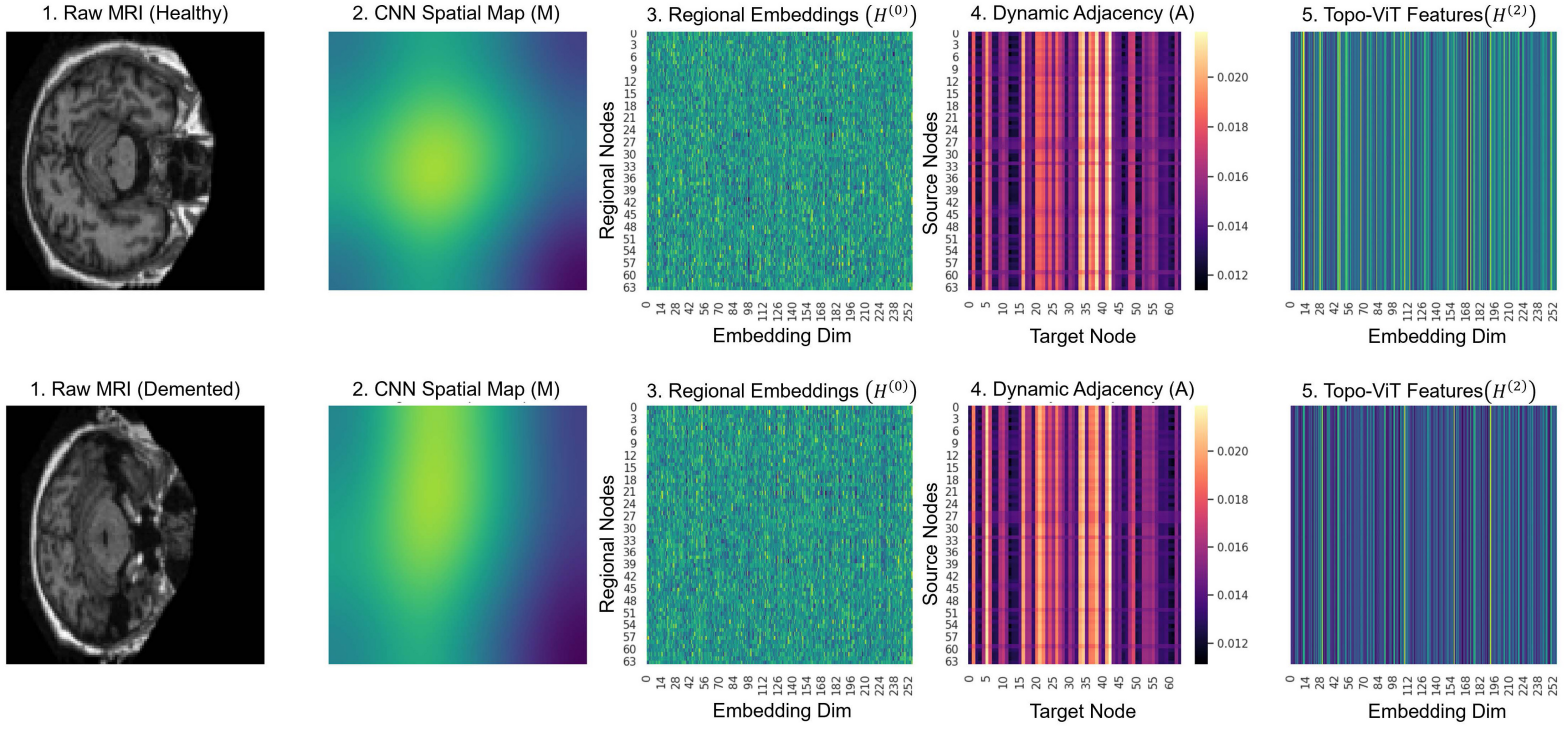
Hierarchical feature transformations from raw structural MRI to topology-aware global representations for healthy and demented samples.

The training process of the proposed hybrid architecture is designed to progressively transform raw, high-dimensional MRI volumes into dense, topology-aware diagnostic representations. The internal progression of this transformation is illustrated in Figure 5, which directly compares processing in a healthy subject with that in a demented subject.

Initially, the raw MRI tensor is processed by the truncated 3D ResNet-18, yielding a localized Convolutional Spatial Map (M). As shown, this stage serves as a volumetric feature extractor, isolating dense structural tissue and highlighting potential cortical atrophy. Subsequently, adaptive pooling collapses this spatial map into 64 distinct 3D regional patches, which are flattened and enriched with positional embeddings to form the Regional Embeddings (*H*^(0)^).

In the critical graph modeling stage, the dynamic GAT processes these regional nodes to computationally infer the patient's individual structural connectivity. This generates the Dynamic Adjacency matrix (*A*), which maps the correlation strength between anatomical regions. In the final stage, this learned patient-specific topology acts as a mathematical bias within the multi-head self-attention mechanism of the Topo-ViT. The resulting Topo-ViT Features (*H*^(2)^) represent a globally reasoned, biologically constrained latent state that directly informs the final dense classification head, ensuring that the diagnosis is governed by both localized tissue deterioration and holistic network disconnection.

### 4.4 Performance analysis of the proposed architecture

**Figure 6:**
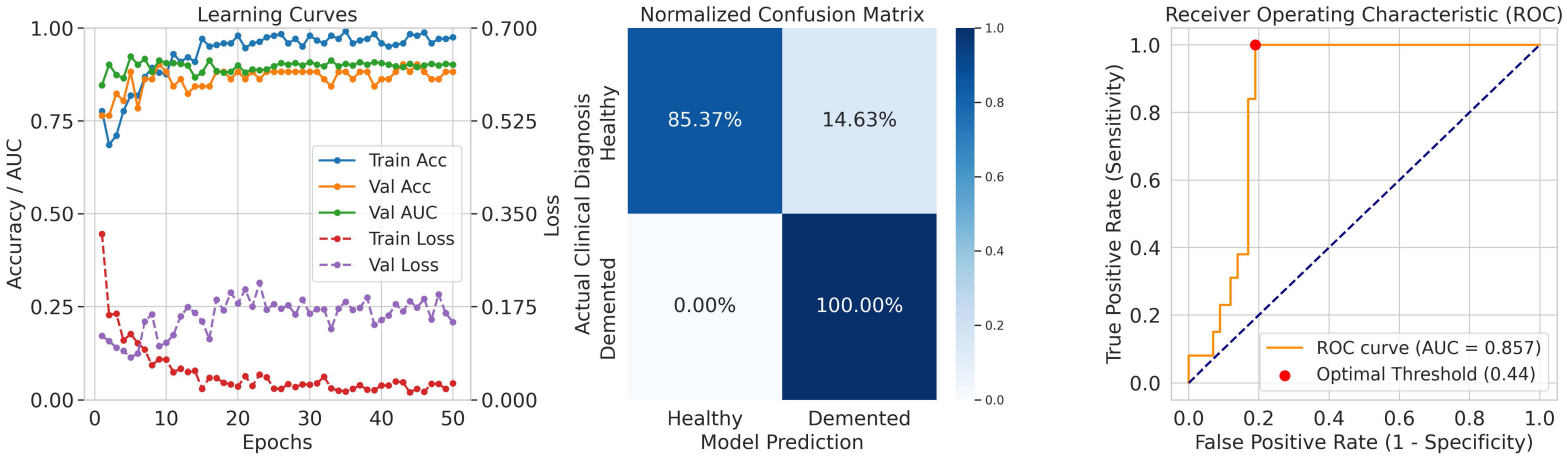
Comprehensive performance evaluation encompassing learning curves, the optimized normalized confusion matrix, and the ROC curve.

The diagnostic performance of the proposed architecture was evaluated on the isolated test set to determine its clinical viability. As depicted in the learning curves in Figure 6, the model demonstrates stable performance over 50 epochs. The validation loss and accuracy closely track the training metrics, indicating effective regularization, stable weight optimization, and no evidence of overfitting. Furthermore, the Receiver Operating Characteristic (ROC) curve yields an Area Under the Curve (AUC) of 0.857, indicating strong discriminative performance across all classification thresholds. By applying Youden's J statistic to the ROC curve, an optimal diagnostic threshold of 0.44 was empirically identified. When using this optimized threshold, the normalized confusion matrix shows high sensitivity, correctly identifying 100% of demented cases in the test cohort, with a specificity of 85.37% for structurally healthy subjects.

These results directly support the study objective of modeling Alzheimer's disease as a network-level disorder. The strong ROC-AUC and high sensitivity indicate that integrating topology-aware attention enables the model to effectively capture both localized atrophy and global structural disconnection patterns.

**Figure 7:**
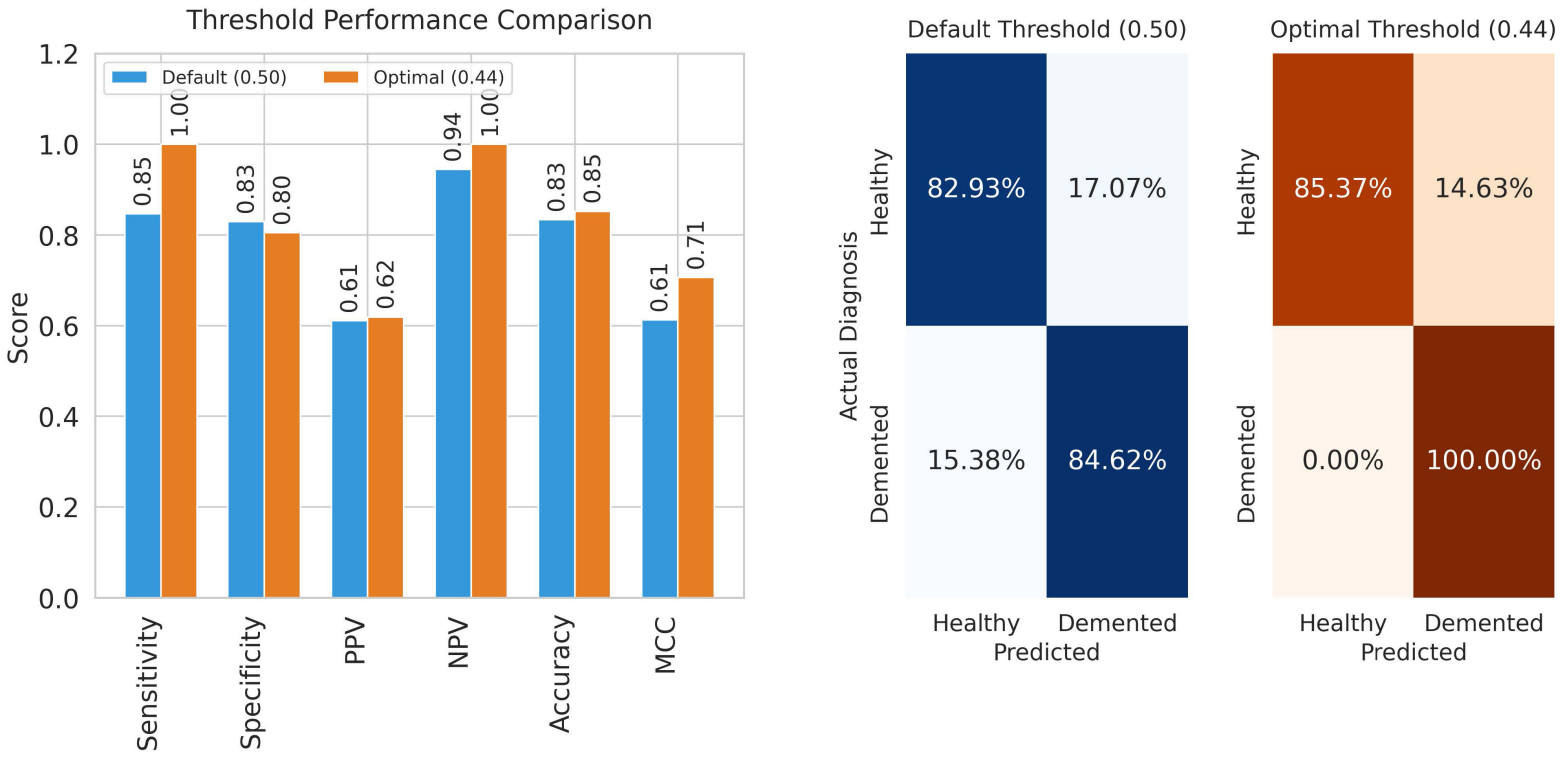
Comparative analysis of clinical classification metrics between the default diagnostic threshold and the statistically optimized threshold.

**Figure 8:**
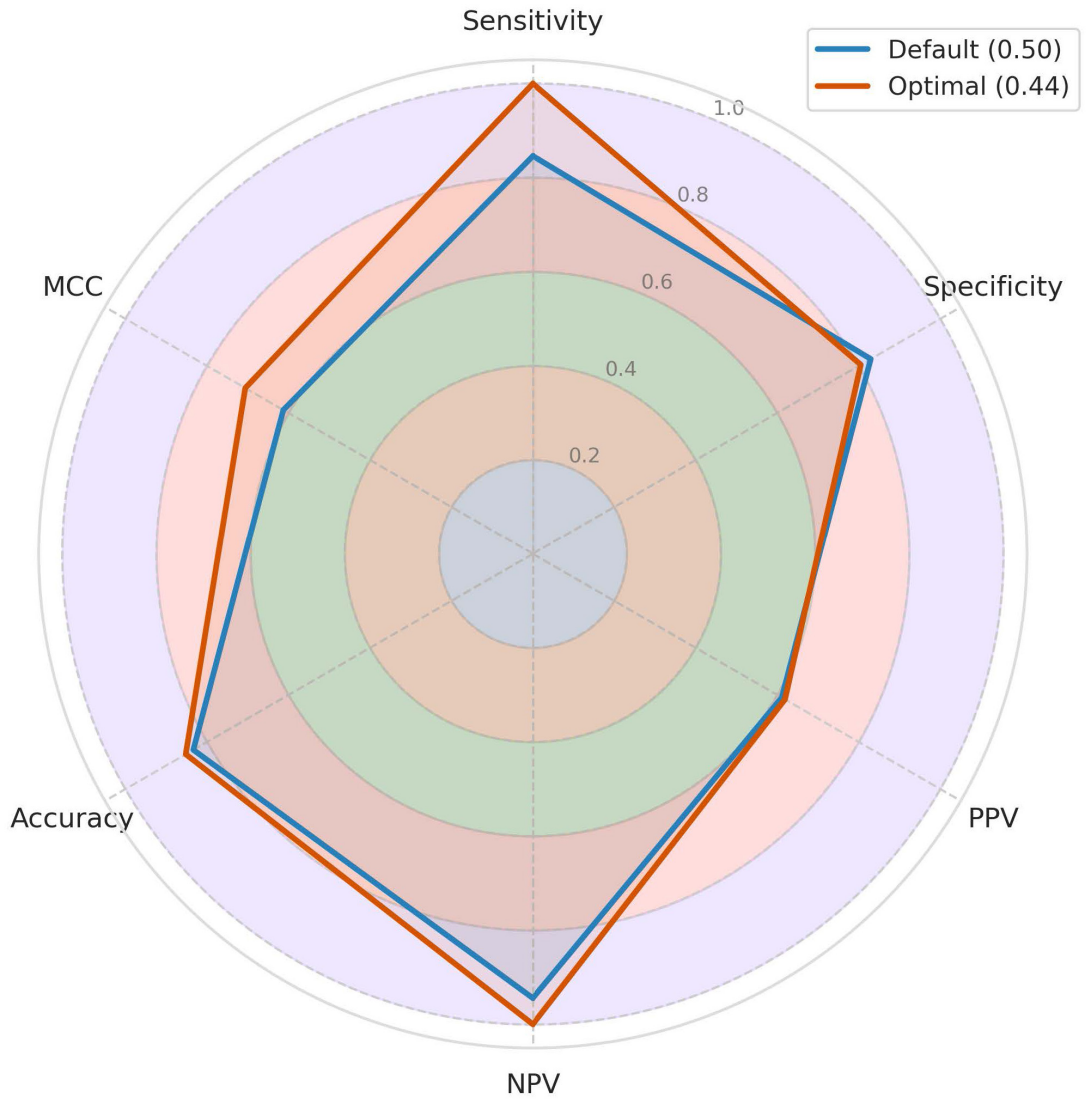
Multivariable spider chart visualizing the performance metric shifts resulting from diagnostic threshold optimization.

To further contextualize the architecture's clinical utility, a comparative analysis was conducted between the standard default probability threshold of 0.50 and the optimized threshold of 0.44. As illustrated by the performance comparison bar chart in Figure 7 and the multivariable spider chart in Figure 8, threshold optimization significantly enhances the network's diagnostic safety profile. Specifically, lowering the decision boundary to 0.44 maximizes sensitivity from 85% to 100% and improves the Negative Predictive Value (NPV) to 1.00. This configuration ensures that no demented patients in the test set are misclassified as healthy, yielding zero false negatives, which is a critical requirement for early screening systems. While this conservative tuning results in a minor reduction in specificity from 83% to 80%, the overall diagnostic accuracy increases to 85%. Furthermore, the Matthews Correlation Coefficient (MCC), a highly robust metric specifically designed for imbalanced datasets, improves substantially from 0.61 to 0.71, affirming the overall superiority of the optimized configuration.

**Figure 9:**
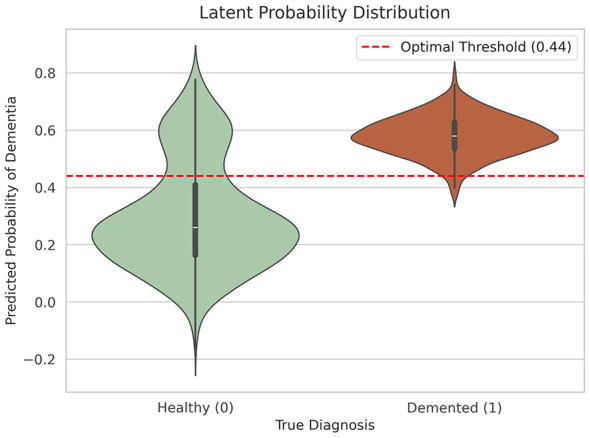
Violin plot illustrating the distribution of the network's predicted dementia probabilities stratified by true clinical diagnosis, with the optimal diagnostic threshold highlighted.

The model's predictive confidence is further clarified by analyzing the latent probability distribution of the test samples, visualized as a violin plot in Figure 9. The clear separation between the probability density masses of the healthy and demented cohorts indicates the effectiveness of the topology-aware representations. The healthy cohort's predictions are tightly clustered in the lower probability range, whereas the demented cohort shows a tightly clustered distribution that peaks significantly higher on the scale. The superimposed optimal threshold line at 0.44 clearly shows that the decision boundary effectively captures the lower tail of the demented distribution, successfully prioritizing maximal sensitivity while maintaining robust clinical discrimination.

### 4.5 Computational performance of the proposed architecture

To ensure the clinical deployability of the proposed hybrid architecture, a rigorous computational performance evaluation was conducted under realistic inference scenarios. Rather than utilizing synthetically generated dummy tensors, the profiling sequence explicitly sampled a genuine 3D structural MRI volume from the validation dataset to ensure valid spatial distributions and representative computational loads. The assessment was executed on an NVIDIA T4 GPU. To achieve sub-millisecond precision, native CUDA timing events were systematically integrated around each distinct architectural block, avoiding the inherent latency inaccuracies associated with standard CPU-bound timers. Furthermore, the core computational complexity was accurately quantified using the *fvcore* diagnostic library to extract the precise floating-point macro-architecture footprint.

The outcomes of this comprehensive profiling are detailed in Table 2. An analysis of the memory footprint shows that the complete network contains 33.94 million trainable parameters, resulting in a model file size of approximately 129.47 MB. Notably, the parameter distribution is heavily skewed toward the foundational stage; the 3D CNN backbone accounts for 97.7% of the total parameter count. In stark contrast, the newly introduced non-Euclidean reasoning modules, the GAT and the Topo-ViT, are lightweight, using only 0.6% and 1.6% of the network's total parameters, respectively. During a standard single-batch inference pass, the peak active Video Random Access Memory (VRAM) allocation remains at 260.34 MB. This memory footprint confirms that the architecture can be seamlessly integrated into legacy hospital IT infrastructure or standard clinical workstations without requiring massive, specialized GPU clusters.

**Table 2:** Computational performance analysis of the proposed design.

Category	Metric	Value
Memory & parameters	Total Parameters	33.94 M
	Trainable Parameters	33.94 M
	Model File Size	~129.47 MB
	CNN Stage Params	33.17 M (97.7%)
	GAT Stage Params	0.21 M (0.6%)
	ViT Stage Params	0.53 M (1.6%)
	Classifier Params	0.03 M (0.1%)
Computational complexity	Theoretical MACs	~18.5 GMACs
	Dominant Component	3D CNN
Runtime memory	Peak VRAM (Batch=1)	260.34 MB
Inference performance	Avg Latency	45.61 ms ± 0.41 ms
	99th Percentile Latency	46.67 ms
	Throughput	21.92 volumes/sec
	Compute Efficiency	405.58 GMACs/sec
Stage bottleneck	Stage 1 (3D CNN)	44.82 ms (99.2%)
	Stage 2 (GAT)	0.16 ms (0.4%)
	Stage 3 (ViT)	0.16 ms (0.4%)
	Classifier	0.03 ms (0.1%)

From an execution speed perspective, the network demonstrates high computational efficiency. Operating at a theoretical computational complexity of approximately 18.5 Giga Multiply-Accumulate Operations (GMACs), the architecture achieves an average end-to-end inference latency of just 45.61 milliseconds per patient scan. The narrow standard deviation and a 99th-percentile latency of 46.67 milliseconds underscore the high stability and predictability of the feed-forward pass. This execution speed translates to a processing throughput of nearly 22 full 3D volumes per second. The stage-by-stage bottleneck analysis reveals the computational efficiency of the topological advancements. The volumetric feature extraction performed by the 3D CNN accounts for 99.2% of the processing time (44.82 milliseconds). The subsequent mathematical inference of patient-specific topologies via the GAT, paired with the structural modulation of global attention via the Topo-ViT, collectively incur a minimal computational overhead of 0.32 milliseconds. This demonstrates that architecture achieves network-level reasoning without incurring detrimental latency overhead, making it a computationally scalable framework for high-throughput radiological environments.

This computational efficiency further supports the study's objective of developing a clinically deployable diagnostic system, demonstrating that incorporating topology-aware and graph-based reasoning does not compromise real-time applicability.

### 4.6 Biological validation and statistical robustness

An important consideration in evaluating the proposed framework is the biological validity of the dynamically inferred, patient-specific structural connectivity graphs, which operate independently of predefined anatomical atlases. To assess the stability of these connectivity patterns under scanner noise and across varying preprocessing environments, a whole-cohort perturbation stability test was conducted. By introducing controlled input perturbations, the average graph structural similarity (measured via Pearson correlation) across the test subjects was 0.7368. This metric demonstrates a high degree of topological resilience, confirming that the inferred networks capture stable anatomical structures rather than arbitrary noise.

**Figure 10:**
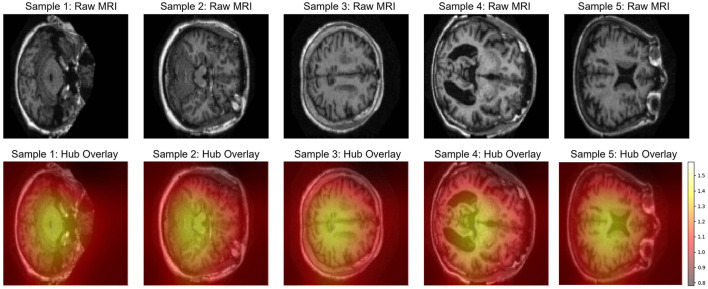
Population-level biological validity mapping illustrating the alignment of dynamic graph network hubs with key anatomical regions of interest in the demented patient cohort.

Furthermore, a population-level biological validity mapping was conducted to assess how these dynamic patterns align with clinically established biomarkers of Alzheimer's disease. By aggregating the topological graphs generated exclusively from the demented test cohort, spatial overlays of network connectivity hubs were produced. As shown in Figure 10, the mathematically derived network hubs align directly with physical regions of interest that are associated with cognitive decline and neurodegeneration. This spatial mapping confirms that architecture's network-level reasoning is grounded in actual biological pathology.

This observation directly supports the study objective of achieving biologically coherent diagnostic inference, confirming that the proposed patient-specific connectivity modeling captures meaningful anatomical relationships rather than spurious correlations.

To substantiate the statistical rigor of the reported performance metrics and confirm that the results are not simply the product of an optimal dataset split, a comprehensive bootstrapping analysis was conducted. Conducting 1000 bootstrap iterations on the test predictions established a 95% confidence interval for the ROC-AUC ranging from 0.7552 to 0.9493. This narrow interval rigorously validates the model's performance gains as robust and clinically significant.

**Figure 11:**
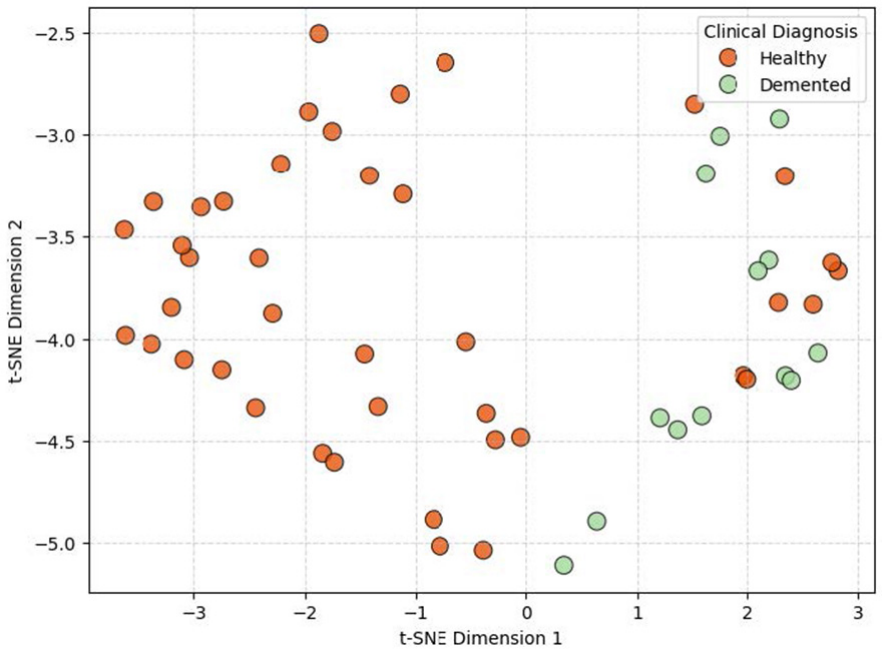
A t-SNE projection of the final topology-aware latent space, demonstrating clear manifold separation between healthy and demented subjects.

Finally, the representational capability of the hybrid architecture was analyzed by projecting the high-dimensional latent space into a two-dimensional plane using t-Distributed Stochastic Neighbor Embedding (t-SNE). The resulting t-SNE projection, visualized in Figure 11, illustrates a clear separation between the healthy and demented clinical classes. This explicit clustering provides empirical evidence that the integrated topology-aware components successfully differentiate the complex, large-scale deterioration patterns inherent to Alzheimer's disease.

### 4.7 Ablation study

To evaluate the contribution and efficiency of the proposed hybrid architecture, a comprehensive ablation study was conducted. This analysis examined the network to isolate and evaluate the individual contributions of deep volumetric feature extraction, patient-specific graph routing, and global transformer-based reasoning. The experimental configurations designed for this study are outlined in Table 3. Five unique architectural scenarios were tested to establish a baseline and progressively integrate the advanced topological components.

**Table 3:** Architectural configurations for ablation study.

Scenario	3D CNN backbone	GAT	Topo-ViT	Core purpose of the experiment
1. Baseline (3D CNN only)	Active	None	Inactive	Establishes the baseline performance of standard, localized volumetric feature extraction without global or topological reasoning.
2. CNN + Topo-ViT	Active	None	Active	Isolates the impact of standard global self-attention mechanisms without structural graph guidance.
3. CNN + Dyn-GAT	Active	Dynamic	Inactive	Isolates the capability of the patient-specific network routing (message passing) without transformer-based long-range reasoning.
4. CNN + Static-GAT + Topo-ViT	Active	Static (Atlas)	Active	Evaluates the performance of the full pipeline when restricted to a fixed, population-level structural prior rather than individualized networks.
5. Full Proposed Model	Active	Dynamic	Active	The complete architecture.

**Figure 12:**
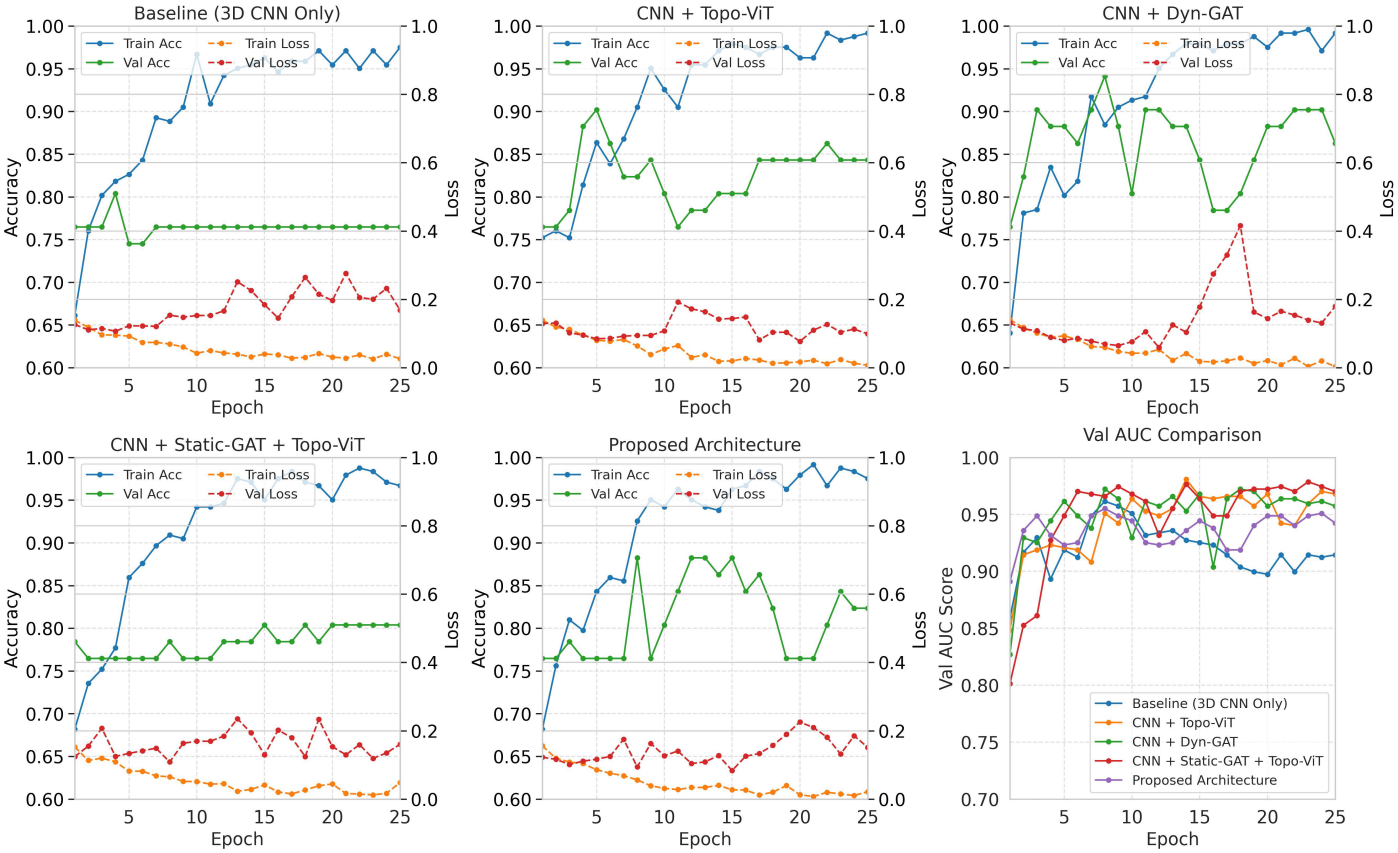
Learning curves and validation AUC trajectories across the five architectural configurations evaluated in the ablation study.

The clinical performance metrics associated with each configuration highlight the synergistic impact of the proposed components. The learning-curve and validation AUC trajectories across the five scenarios are shown in Figure 12. The baseline CNN model, which relies entirely on localized volumetric features, shows lower convergence stability and achieves an AUC of 0.8330. Introducing only the Vision Transformer (Scenario 2) or only the Dynamic GAT (Scenario 3) yields isolated performance shifts, but neither configuration optimally captures the full spectrum of Alzheimer's pathology. Furthermore, Scenario 4 demonstrates that using a static, atlas-based adjacency matrix limits the network performance, showing that generic population priors fail to capture the individualized nature of neurodegenerative decline.

**Figure 13:**
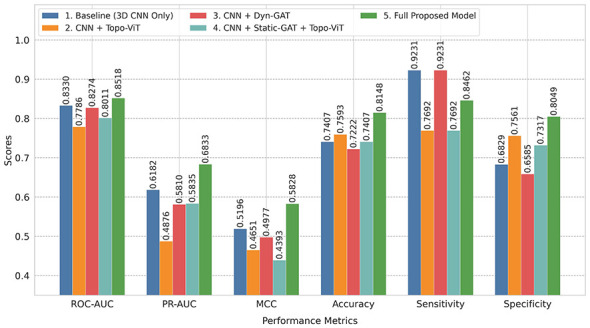
Comprehensive comparison of clinical evaluation metrics across all ablated architectural configurations.

As summarized in the comprehensive performance chart in Figure 13, the fully integrated proposed model (Scenario 5) outperforms all ablated variants. By mathematically coupling dynamic graph generation with the transformer's global self-attention mechanism, the full model achieves the highest overall accuracy of 81.48% and the highest precision-recall area under the curve (PR-AUC) of 0.6833. Notably, the proposed architecture increases specificity to 0.8049 while maintaining robust sensitivity, yielding an optimal MCC of 0.5828. This demonstrates that global reasoning is most effective when explicitly constrained by dynamically learned, patient-specific physical topologies.

Crucially, this improvement in clinical discriminability does not impose an excessive computational burden. The computational performance of the ablation scenarios is detailed in Table 4.

**Table 4:** Computational performance of the ablation study.

Scenario	Params (M)	Size (MB)	GMACs	Peak VRAM (MB)	Latency (ms)	Throughput (FPS)
1. Baseline (3D CNN Only)	33.35	127.2	18.5	258.1	49.04	20.4
2. CNN + Topo-ViT	33.87	129.2	18.5	268.5	50.01	20.0
3. CNN + Dyn-GAT	33.41	127.5	18.5	266.7	49.99	20.0
4. CNN + Static-GAT + Topo-ViT	33.94	129.5	18.5	268.8	50.45	19.8
5. Full Proposed Model	33.94	129.5	18.5	268.7	51.14	19.6

Transitioning from the foundational baseline model to the fully integrated topology-aware architecture incurs only a minimal overhead. The total number of trainable parameters increases from 33.35 million to 33.94 million, adding approximately 2.3 MB of memory, a small increase. In active deployment, the peak VRAM remains nearly identical, ensuring the model remains deployment-friendly. Most importantly, the sophisticated network-level operations introduce a minimal latency penalty of precisely 2.1 milliseconds, causing the processing throughput to drop insignificantly from 20.4 to 19.6 volumes per second. This demonstrates that the proposed architectural enhancements achieve state-of-the-art diagnostic accuracy while fully preserving the high computational efficiency required for rapid clinical inference.

Overall, these findings validate the central study objective that optimal diagnostic performance is achieved through the joint integration of volumetric feature extraction, dynamic connectivity modeling, and topology-guided global attention, rather than relying on any single component in isolation.

## 5 Conclusion

This study presented a topology-aware hybrid DL framework for Alzheimer's disease diagnosis using structural MRI, designed to model the disease as a large-scale network-disconnection process. By integrating a 3D convolutional backbone, a dynamic graph attention network, and a topology-biased vision transformer, the proposed architecture effectively captures both localized morphometric alterations and global structural connectivity patterns. Unlike conventional approaches that rely on 2D representations or predefined atlases, the framework learns patient-specific connectivity directly from volumetric data and incorporates this information into global attention mechanisms.

Comprehensive experimental evaluation demonstrates that the proposed model achieves robust diagnostic performance under strict subject-level data segregation, ensuring clinically reliable generalization. The results further indicate that topology-guided attention enhances discriminative capability compared with baseline CNN and hybrid architectures. In particular, achieving high sensitivity while maintaining competitive specificity highlights the model's potential for early-stage screening applications, where minimizing false negatives is critical. Additionally, the stability of the learned connectivity patterns and the clear separation of latent representations suggest that the framework captures biologically meaningful disease signatures rather than dataset-specific artifacts.

Despite these promising results, several limitations remain. The current evaluation relies on a single dataset with a moderate sample size, which may limit generalizability across diverse populations and imaging protocols. Furthermore, the framework focuses exclusively on structural MRI and excludes complementary modalities, such as PET imaging or clinical biomarkers, that could further enhance diagnostic accuracy.

Future work will focus on extending the proposed framework to larger, multi-center datasets to validate its robustness across heterogeneous clinical settings. Incorporating multimodal data, such as functional imaging and cognitive assessments, may provide a more comprehensive characterization of disease progression. Additionally, exploring longitudinal modeling could enable early prediction of disease trajectories and support personalized clinical decision-making. Finally, efforts to optimize lightweight models and deploy them in real-world clinical environments will be essential to translate the proposed approach into practical diagnostic tools.

## Data Availability

The data used in this study are publicly available. The original structural MRI data were obtained from the Open Access Series of Imaging Studies (OASIS-1 dataset, available at https://sites.wustl.edu/oasisbrains/home/oasis-1/). A processed version of the OASIS-1 dataset, derived from the original scans and made available for machine learning research, was accessed through Kaggle at https://www.kaggle.com/datasets/ninadaithal/imagesoasis. The code and Jupyter notebooks developed to implement and evaluate the proposed topology-aware hybrid graph-transformer framework are available from the corresponding author upon reasonable request for academic and research purposes.
